# 2-[4,5-Diphenyl-2-(pyridin-2-yl)-1*H*-imidazol-1-yl]-3-phenyl­propan-1-ol

**DOI:** 10.1107/S1600536812018703

**Published:** 2012-05-12

**Authors:** Liangru Yang, Yongmei Xiao, Kun He, Jinwei Yuan, Pu Mao

**Affiliations:** a School of Chemistry and Chemical Engineering, Henan University of Technology, Zhengzhou 450001, People’s Republic of China

## Abstract

In the title compound, C_29_H_25_N_3_O, the central imidazole ring forms dihedral angles of 64.7 (3), 33.5 (3) and 81.2 (2)° with the pyridyl and two phenyl substituents, respectively. An intra­molecular C—H⋯N hydrogen bond is observed. In the crystal, O—H⋯N and C—H⋯O hydrogen bonds link the mol­ecules into chains parallel to the *a* axis.

## Related literature

For the synthesis and properties of chiral ionic liquids, see: Ding & Armstrong (2005 ▶); Bwambok *et al.* (2008 ▶); Mao *et al.* (2010 ▶). For a related structure, see: Xiao *et al.* (2012 ▶).
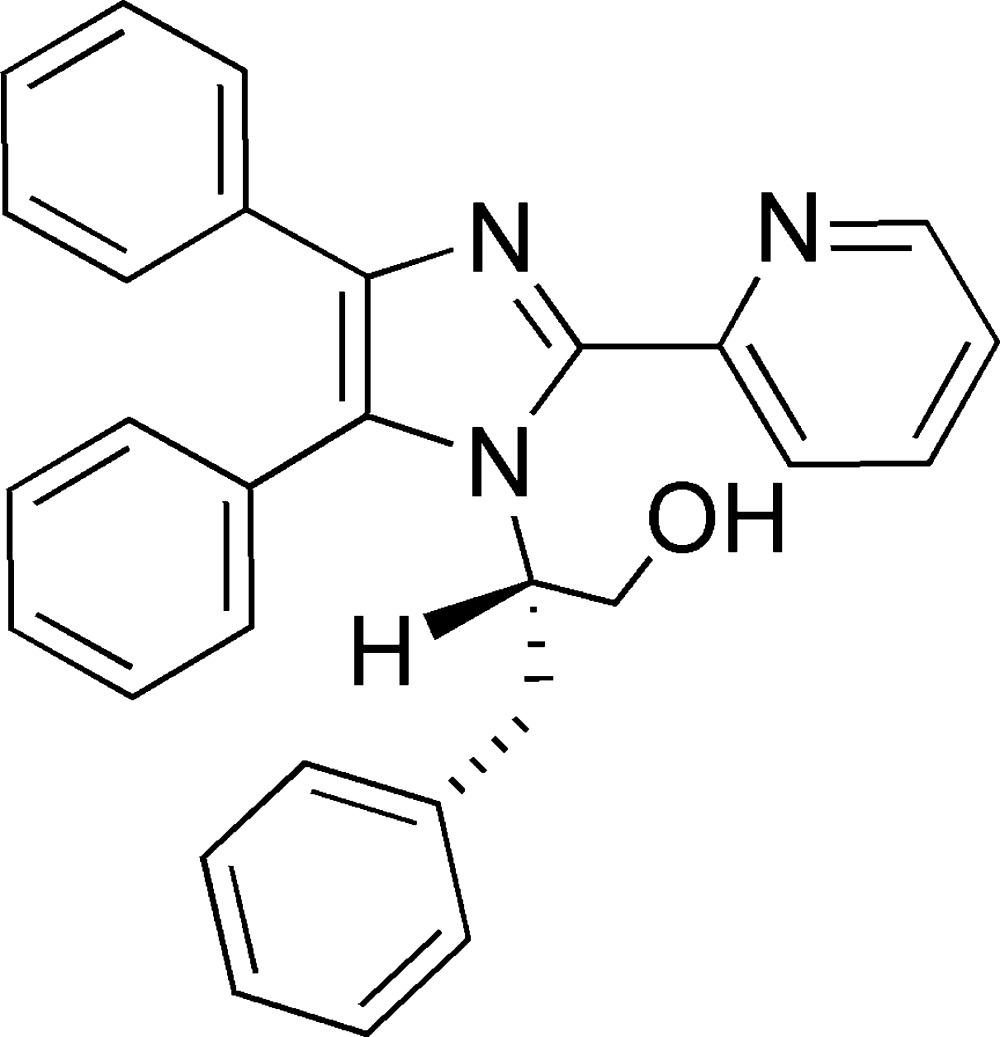


## Experimental

### 

#### Crystal data

C_29_H_25_N_3_O
*M* *_r_* = 431.52
Orthorhombic, 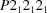

*a* = 9.2695 (4) Å
*b* = 15.8818 (6) Å
*c* = 16.0498 (6) Å
*V* = 2362.79 (16) Å^3^
*Z* = 4
Cu *K*α radiation
μ = 0.58 mm^−1^
*T* = 291 K
0.38 × 0.28 × 0.25 mm

#### Data collection

Oxford Diffraction Xcalibur Eos Gemini diffractometer
Absorption correction: multi-scan (*CrysAlis PRO*; Agilent, 2011 ▶) *T*_min_ = 0.809, *T*_max_ = 0.868
9413 measured reflections
4442 independent reflections
3981 reflections with *I* > 2σ(*I*)
*R*_int_ = 0.029

#### Refinement

*R*[*F* ^2^ > 2σ(*F* ^2^)] = 0.038
*wR*(*F* ^2^) = 0.102
*S* = 1.04
4442 reflections
311 parameters
H atoms treated by a mixture of independent and constrained refinement
Δρ_max_ = 0.12 e Å^−3^
Δρ_min_ = −0.13 e Å^−3^
Absolute structure: Flack (1983 ▶), 1871 Friedel pairs
Flack parameter: 0.2 (4)

### 

Data collection: *CrysAlis PRO* (Agilent, 2011 ▶); cell refinement: *CrysAlis PRO*; data reduction: *CrysAlis PRO*; program(s) used to solve structure: *SHELXS97* (Sheldrick, 2008 ▶); program(s) used to refine structure: *SHELXL97* (Sheldrick, 2008 ▶); molecular graphics: *OLEX2* (Dolomanov *et al.*, 2009 ▶); software used to prepare material for publication: *OLEX2*.

## Supplementary Material

Crystal structure: contains datablock(s) I, global. DOI: 10.1107/S1600536812018703/rz2739sup1.cif

Structure factors: contains datablock(s) I. DOI: 10.1107/S1600536812018703/rz2739Isup2.hkl

Supplementary material file. DOI: 10.1107/S1600536812018703/rz2739Isup3.cml

Additional supplementary materials:  crystallographic information; 3D view; checkCIF report

## Figures and Tables

**Table 1:** Hydrogen-bond geometry (Å, °)

*D*—H⋯*A*	*D*—H	H⋯*A*	*D*⋯*A*	*D*—H⋯*A*
C27—H27*A*⋯N3	0.97	2.42	3.277 (2)	146
C22—H22⋯O1^i^	0.93	2.54	3.350 (3)	146
O1—H1⋯N1^ii^	0.85 (3)	1.94 (3)	2.789 (2)	174 (3)

Symmetry codes: (i) 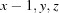; (ii) 
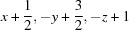
.

**Fig. 1.**
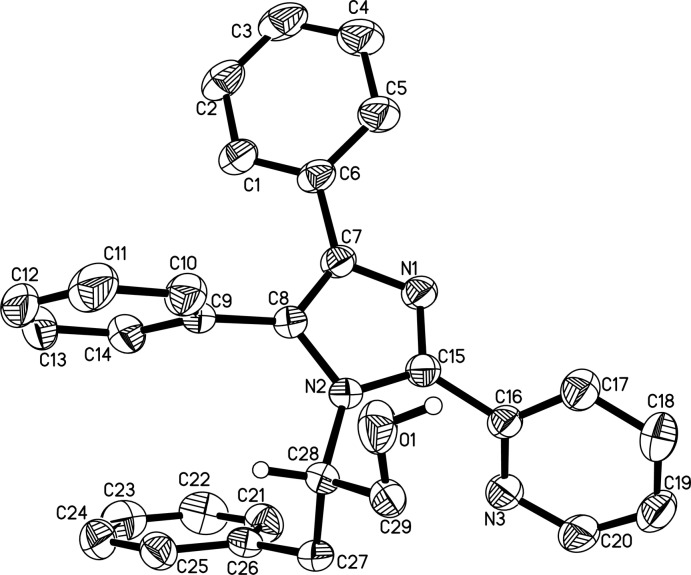
The molecular structure of the title compound showing 30% probability displacement ellipsoids. Hydrogen atoms are omitted for clarity.

## supplementary crystallographic information

### Comment

Our group is interested in the synthesis of chiral imidazole derivatives from natural precursors through a convenient four component–one pot synthetic protocol using an aldehyde, glyoxal, ammonia, and an amine (Mao *et al.*, 2010). During our studies we observed that by carefully choosing the four components, a number of different imidazole derivatives could be obtained easily (Xiao *et al.*, 2012). The condensation of *L*-phenylalaninol, dibenzoyl, 2-formyl pyridine and ammonium acetate afforded the title compound. This compound may serve as a starting material for the research of imidazolium based chiral ionic liquids in catalysis, chiral recognization and separation (Ding & Armstrong, 2005; Bwambok *et al.*, 2008).

The molecular structure of the title compound is shown in Figure 1. As expected, the imidazole core (N1, C7, C8, N2, C15) is essentially planar, featuring an average deviation smaller than 0.6 (2) Å. The dihedral angle between the pyridyl and imidazole rings is 64.7 (3) °, and the dihedral angles between the two phenyl substituents and the imidazole ring are 33.5 (3)° and 81.2 (2)°, respectively. The chiral C28 atom maintains the *S* configuration of the *L*-phenylalaninol. The molecular conformation is enforced by an intramolecular C—H···N hydrogen bond (Table 1). In the crystal structure, molecules are linked by intermolecular O–H···N and C—H···O hydrogen bonds into chains parallel to the *a* axis.

### Experimental

To a solution of *L*-phenylalaninol (15.1 g, 0.1 mol) in MeOH (50 ml) in an ice-bath, molar equivalents of dibenzoyl, 2-formyl pyridine and ammonium acetate were added. The mixture was kept stirring in the ice-bath until all the solids were dissolved before being heated to 60°C for 5 h. The mixture was then cooled to r.t. and the solvent was removed by evaporation. The residue was washed with H_2_O to obtain the crude product. Crystallization of the crude product in EtOH afforded colourless crystals of the title compound.

### Refinement

The H atoms associated to the hydroxy group and to the C29 methylene group were located in a difference Fourier map and refined freely. All other H atoms were positioned geometrically and refined using a riding model, with C—H = 0.93-0.98 Å, and with *U*_iso_(H) = 1.2 *U*_eq_(C).

### Crystal data

**Table:**

C_29_H_25_N_3_O	*F*(000) = 912
*M**_r_* = 431.52	*D*_x_ = 1.213 Mg m^−^^3^
Orthorhombic, *P*2_1_2_1_2_1_	Cu *K*α radiation, λ = 1.54178 Å
Hall symbol: P 2ac 2ab	Cell parameters from 3448 reflections
*a* = 9.2695 (4) Å	θ = 3.9–70.3°
*b* = 15.8818 (6) Å	µ = 0.58 mm^−^^1^
*c* = 16.0498 (6) Å	*T* = 291 K
*V* = 2362.79 (16) Å^3^	Prismatic, colourless
*Z* = 4	0.38 × 0.28 × 0.25 mm

### Data collection

**Table:**

Oxford Diffraction Xcalibur Eos Gemini diffractometer	4442 independent reflections
Radiation source: fine-focus sealed tube	3981 reflections with *I* > 2σ(*I*)
Graphite monochromator	*R*_int_ = 0.029
ω scans	θ_max_ = 70.4°, θ_min_ = 3.9°
Absorption correction: multi-scan (*CrysAlis PRO*; Agilent, 2011)	*h* = −11→6
*T*_min_ = 0.809, *T*_max_ = 0.868	*k* = −19→19
9413 measured reflections	*l* = −19→18

### Refinement

**Table:**

Refinement on *F*^2^	Hydrogen site location: inferred from neighbouring sites
Least-squares matrix: full	H atoms treated by a mixture of independent and constrained refinement
*R*[*F*^2^ > 2σ(*F*^2^)] = 0.038	*w* = 1/[σ^2^(*F*_o_^2^) + (0.0468*P*)^2^ + 0.1152*P*] where *P* = (*F*_o_^2^ + 2*F*_c_^2^)/3
*wR*(*F*^2^) = 0.102	(Δ/σ)_max_ < 0.001
*S* = 1.04	Δρ_max_ = 0.12 e Å^−^^3^
4442 reflections	Δρ_min_ = −0.13 e Å^−^^3^
311 parameters	Extinction correction: *SHELXL97* (Sheldrick, 2008), Fc^*^=kFc[1+0.001xFc^2^λ^3^/sin(2θ)]^-1/4^
0 restraints	Extinction coefficient: 0.0043 (3)
Primary atom site location: structure-invariant direct methods	Absolute structure: Flack (1983), 1871 Friedel pairs
Secondary atom site location: difference Fourier map	Flack parameter: 0.2 (4)

### Special details

**Table:**

Geometry. All e.s.d.'s (except the e.s.d. in the dihedral angle between two l.s. planes) are estimated using the full covariance matrix. The cell e.s.d.'s are taken into account individually in the estimation of e.s.d.'s in distances, angles and torsion angles; correlations between e.s.d.'s in cell parameters are only used when they are defined by crystal symmetry. An approximate (isotropic) treatment of cell e.s.d.'s is used for estimating e.s.d.'s involving l.s. planes.
Refinement. Refinement of *F*^2^ against ALL reflections. The weighted *R*-factor *wR* and goodness of fit *S* are based on *F*^2^, conventional *R*-factors *R* are based on *F*, with *F* set to zero for negative *F*^2^. The threshold expression of *F*^2^ > σ(*F*^2^) is used only for calculating *R*-factors(gt) *etc*. and is not relevant to the choice of reflections for refinement. *R*-factors based on *F*^2^ are statistically about twice as large as those based on *F*, and *R*- factors based on ALL data will be even larger.

### Fractional atomic coordinates and isotropic or equivalent isotropic displacement parameters (Å^2^)

**Table:**

	*x*	*y*	*z*	*U*_iso_*/*U*_eq_	
O1	0.74800 (17)	0.67325 (12)	0.63461 (10)	0.0746 (4)	
H1	0.788 (3)	0.6736 (19)	0.5871 (18)	0.094 (9)*	
N1	0.38909 (18)	0.81423 (9)	0.51834 (9)	0.0550 (4)	
N2	0.45320 (16)	0.73451 (8)	0.62480 (8)	0.0463 (3)	
N3	0.3134 (2)	0.60522 (11)	0.49389 (10)	0.0667 (5)	
C1	0.3385 (3)	1.00699 (12)	0.64156 (14)	0.0632 (5)	
H1A	0.3170	0.9829	0.6929	0.076*	
C2	0.3175 (3)	1.09285 (14)	0.63001 (17)	0.0765 (7)	
H2	0.2801	1.1254	0.6731	0.092*	
C3	0.3515 (4)	1.12968 (14)	0.55573 (18)	0.0897 (9)	
H3	0.3390	1.1873	0.5484	0.108*	
C4	0.4043 (4)	1.08104 (15)	0.49205 (18)	0.0922 (8)	
H4	0.4270	1.1059	0.4413	0.111*	
C5	0.4241 (3)	0.99523 (14)	0.50254 (15)	0.0744 (6)	
H5	0.4599	0.9629	0.4588	0.089*	
C6	0.3908 (2)	0.95713 (11)	0.57826 (12)	0.0559 (5)	
C7	0.4089 (2)	0.86522 (10)	0.58664 (11)	0.0507 (4)	
C8	0.44924 (19)	0.81699 (10)	0.65331 (10)	0.0462 (4)	
C9	0.5020 (2)	0.84066 (10)	0.73772 (10)	0.0499 (4)	
C10	0.6491 (3)	0.84668 (14)	0.75092 (14)	0.0680 (5)	
H10	0.7133	0.8351	0.7079	0.082*	
C11	0.7002 (4)	0.87002 (17)	0.82853 (18)	0.0939 (9)	
H11	0.7990	0.8748	0.8372	0.113*	
C12	0.6071 (5)	0.88607 (16)	0.89254 (16)	0.1047 (13)	
H12	0.6425	0.9013	0.9446	0.126*	
C13	0.4622 (4)	0.87974 (17)	0.87992 (14)	0.0975 (10)	
H13	0.3989	0.8909	0.9235	0.117*	
C14	0.4087 (3)	0.85679 (14)	0.80277 (13)	0.0712 (6)	
H14	0.3096	0.8522	0.7947	0.085*	
C15	0.4153 (2)	0.73696 (11)	0.54294 (10)	0.0496 (4)	
C16	0.4123 (2)	0.66518 (10)	0.48335 (10)	0.0503 (4)	
C17	0.5080 (3)	0.66655 (14)	0.41749 (13)	0.0661 (5)	
H17	0.5735	0.7105	0.4115	0.079*	
C18	0.5044 (3)	0.60158 (16)	0.36082 (15)	0.0778 (6)	
H18	0.5682	0.6007	0.3161	0.093*	
C19	0.4059 (3)	0.53848 (14)	0.37106 (15)	0.0769 (6)	
H19	0.4017	0.4934	0.3342	0.092*	
C20	0.3140 (3)	0.54377 (14)	0.43719 (14)	0.0771 (7)	
H20	0.2462	0.5011	0.4433	0.093*	
C21	0.1277 (2)	0.65894 (16)	0.71789 (14)	0.0702 (5)	
H21	0.1068	0.6486	0.6621	0.084*	
C22	0.0228 (3)	0.69407 (19)	0.7686 (2)	0.0900 (8)	
H22	−0.0677	0.7066	0.7469	0.108*	
C23	0.0523 (3)	0.71040 (17)	0.85053 (19)	0.0858 (8)	
H23	−0.0175	0.7347	0.8845	0.103*	
C24	0.1850 (3)	0.69072 (16)	0.88228 (15)	0.0768 (6)	
H24	0.2050	0.7012	0.9381	0.092*	
C25	0.2892 (2)	0.65539 (14)	0.83191 (13)	0.0620 (5)	
H25	0.3791	0.6423	0.8543	0.074*	
C26	0.2624 (2)	0.63902 (11)	0.74854 (11)	0.0518 (4)	
C27	0.3778 (2)	0.60045 (11)	0.69460 (11)	0.0550 (4)	
H27A	0.3347	0.5818	0.6427	0.066*	
H27B	0.4167	0.5513	0.7226	0.066*	
C28	0.5014 (2)	0.66113 (10)	0.67490 (10)	0.0475 (4)	
H28	0.5348	0.6836	0.7284	0.057*	
C29	0.6303 (2)	0.61828 (12)	0.63495 (11)	0.0552 (4)	
H29A	0.607 (2)	0.5961 (13)	0.5795 (12)	0.054 (5)*	
H29B	0.656 (2)	0.5676 (14)	0.6711 (13)	0.058 (6)*	

### Atomic displacement parameters (Å^2^)

**Table:**

	*U*^11^	*U*^22^	*U*^33^	*U*^12^	*U*^13^	*U*^23^
O1	0.0663 (9)	0.0981 (12)	0.0593 (8)	−0.0147 (9)	0.0172 (7)	−0.0167 (8)
N1	0.0679 (9)	0.0455 (7)	0.0516 (8)	−0.0010 (7)	−0.0131 (7)	0.0021 (6)
N2	0.0545 (8)	0.0394 (6)	0.0449 (7)	−0.0044 (6)	0.0003 (6)	0.0021 (5)
N3	0.0840 (12)	0.0588 (9)	0.0571 (9)	−0.0182 (9)	−0.0078 (8)	−0.0028 (8)
C1	0.0696 (13)	0.0496 (9)	0.0703 (12)	−0.0003 (9)	−0.0161 (11)	−0.0032 (9)
C2	0.0835 (15)	0.0513 (11)	0.0947 (17)	0.0112 (11)	−0.0284 (14)	−0.0126 (11)
C3	0.115 (2)	0.0438 (9)	0.110 (2)	0.0078 (12)	−0.0412 (17)	0.0066 (12)
C4	0.131 (2)	0.0600 (13)	0.0858 (16)	−0.0023 (15)	−0.0165 (18)	0.0223 (12)
C5	0.1002 (18)	0.0520 (10)	0.0711 (12)	0.0030 (11)	−0.0102 (13)	0.0109 (9)
C6	0.0619 (12)	0.0431 (8)	0.0626 (10)	−0.0013 (8)	−0.0200 (9)	0.0003 (8)
C7	0.0562 (10)	0.0428 (8)	0.0530 (9)	−0.0027 (8)	−0.0081 (8)	−0.0006 (7)
C8	0.0498 (9)	0.0419 (7)	0.0470 (8)	−0.0029 (7)	0.0014 (7)	0.0001 (6)
C9	0.0668 (10)	0.0383 (7)	0.0446 (8)	−0.0040 (7)	−0.0009 (8)	0.0027 (6)
C10	0.0750 (13)	0.0635 (11)	0.0654 (11)	−0.0066 (10)	−0.0133 (11)	−0.0033 (10)
C11	0.118 (2)	0.0734 (14)	0.0907 (18)	−0.0084 (15)	−0.0504 (17)	−0.0026 (14)
C12	0.199 (4)	0.0574 (12)	0.0574 (13)	−0.0037 (19)	−0.0422 (19)	−0.0018 (10)
C13	0.172 (3)	0.0718 (15)	0.0484 (11)	0.0041 (18)	0.0144 (16)	−0.0023 (10)
C14	0.0922 (16)	0.0635 (11)	0.0578 (11)	−0.0013 (11)	0.0134 (11)	0.0014 (9)
C15	0.0545 (10)	0.0452 (8)	0.0492 (8)	−0.0024 (8)	−0.0041 (8)	0.0019 (7)
C16	0.0595 (10)	0.0438 (8)	0.0478 (8)	0.0027 (8)	−0.0096 (8)	0.0010 (7)
C17	0.0654 (12)	0.0655 (11)	0.0675 (11)	−0.0032 (10)	0.0017 (10)	−0.0087 (9)
C18	0.0777 (14)	0.0837 (15)	0.0721 (13)	0.0122 (13)	0.0085 (12)	−0.0193 (12)
C19	0.1013 (18)	0.0572 (11)	0.0721 (13)	0.0065 (12)	−0.0160 (14)	−0.0179 (10)
C20	0.1032 (19)	0.0589 (11)	0.0693 (13)	−0.0229 (13)	−0.0153 (13)	−0.0068 (10)
C21	0.0683 (13)	0.0761 (13)	0.0663 (12)	−0.0071 (11)	−0.0070 (10)	0.0059 (10)
C22	0.0617 (14)	0.0954 (18)	0.113 (2)	0.0056 (13)	0.0016 (14)	0.0151 (16)
C23	0.0765 (16)	0.0768 (15)	0.1042 (19)	0.0024 (12)	0.0317 (15)	0.0014 (14)
C24	0.0943 (17)	0.0745 (13)	0.0616 (12)	−0.0117 (13)	0.0199 (12)	−0.0041 (10)
C25	0.0652 (12)	0.0650 (11)	0.0557 (10)	−0.0059 (9)	0.0038 (9)	0.0049 (9)
C26	0.0590 (10)	0.0444 (8)	0.0521 (9)	−0.0102 (8)	0.0057 (8)	0.0076 (7)
C27	0.0706 (12)	0.0403 (8)	0.0542 (9)	−0.0067 (8)	0.0078 (9)	0.0018 (7)
C28	0.0596 (10)	0.0407 (7)	0.0423 (7)	0.0000 (7)	0.0018 (7)	0.0049 (6)
C29	0.0674 (11)	0.0531 (9)	0.0452 (8)	0.0070 (9)	0.0046 (8)	0.0036 (8)

### Geometric parameters (Å, º)

**Table:**

O1—H1	0.85 (3)	C13—H13	0.9300
O1—C29	1.397 (3)	C13—C14	1.383 (4)
N1—C7	1.375 (2)	C14—H14	0.9300
N1—C15	1.312 (2)	C15—C16	1.488 (2)
N2—C8	1.388 (2)	C16—C17	1.380 (3)
N2—C15	1.360 (2)	C17—H17	0.9300
N2—C28	1.485 (2)	C17—C18	1.376 (3)
N3—C16	1.333 (3)	C18—H18	0.9300
N3—C20	1.334 (3)	C18—C19	1.366 (4)
C1—H1A	0.9300	C19—H19	0.9300
C1—C2	1.390 (3)	C19—C20	1.364 (4)
C1—C6	1.376 (3)	C20—H20	0.9300
C2—H2	0.9300	C21—H21	0.9300
C2—C3	1.365 (4)	C21—C22	1.385 (4)
C3—H3	0.9300	C21—C26	1.379 (3)
C3—C4	1.371 (4)	C22—H22	0.9300
C4—H4	0.9300	C22—C23	1.368 (4)
C4—C5	1.385 (3)	C23—H23	0.9300
C5—H5	0.9300	C23—C24	1.368 (4)
C5—C6	1.392 (3)	C24—H24	0.9300
C6—C7	1.476 (2)	C24—C25	1.379 (3)
C7—C8	1.368 (2)	C25—H25	0.9300
C8—C9	1.488 (2)	C25—C26	1.386 (3)
C9—C10	1.384 (3)	C26—C27	1.507 (3)
C9—C14	1.380 (3)	C27—H27A	0.9700
C10—H10	0.9300	C27—H27B	0.9700
C10—C11	1.383 (3)	C27—C28	1.530 (2)
C11—H11	0.9300	C28—H28	0.9800
C11—C12	1.366 (5)	C28—C29	1.517 (3)
C12—H12	0.9300	C29—H29A	0.98 (2)
C12—C13	1.361 (5)	C29—H29B	1.02 (2)
			
C29—O1—H1	110 (2)	N3—C16—C15	118.61 (17)
C15—N1—C7	106.63 (14)	N3—C16—C17	123.39 (18)
C8—N2—C28	124.76 (14)	C17—C16—C15	117.91 (17)
C15—N2—C8	106.54 (14)	C16—C17—H17	120.7
C15—N2—C28	128.55 (14)	C18—C17—C16	118.6 (2)
C16—N3—C20	115.7 (2)	C18—C17—H17	120.7
C2—C1—H1A	119.5	C17—C18—H18	120.4
C6—C1—H1A	119.5	C19—C18—C17	119.1 (2)
C6—C1—C2	121.0 (2)	C19—C18—H18	120.4
C1—C2—H2	119.9	C18—C19—H19	121.1
C3—C2—C1	120.3 (2)	C20—C19—C18	117.8 (2)
C3—C2—H2	119.9	C20—C19—H19	121.1
C2—C3—H3	120.3	N3—C20—C19	125.3 (2)
C2—C3—C4	119.5 (2)	N3—C20—H20	117.3
C4—C3—H3	120.3	C19—C20—H20	117.3
C3—C4—H4	119.6	C22—C21—H21	119.4
C3—C4—C5	120.7 (3)	C26—C21—H21	119.4
C5—C4—H4	119.6	C26—C21—C22	121.2 (2)
C4—C5—H5	119.9	C21—C22—H22	120.0
C4—C5—C6	120.3 (2)	C23—C22—C21	120.1 (3)
C6—C5—H5	119.9	C23—C22—H22	120.0
C1—C6—C5	118.20 (19)	C22—C23—H23	120.2
C1—C6—C7	122.82 (19)	C24—C23—C22	119.6 (3)
C5—C6—C7	118.96 (19)	C24—C23—H23	120.2
N1—C7—C6	119.65 (16)	C23—C24—H24	119.8
C8—C7—N1	109.29 (15)	C23—C24—C25	120.3 (2)
C8—C7—C6	131.02 (16)	C25—C24—H24	119.8
N2—C8—C9	121.99 (15)	C24—C25—H25	119.4
C7—C8—N2	106.12 (14)	C24—C25—C26	121.1 (2)
C7—C8—C9	131.32 (15)	C26—C25—H25	119.4
C10—C9—C8	118.72 (18)	C21—C26—C25	117.7 (2)
C14—C9—C8	122.0 (2)	C21—C26—C27	122.11 (18)
C14—C9—C10	119.3 (2)	C25—C26—C27	120.24 (19)
C9—C10—H10	120.2	C26—C27—H27A	108.9
C11—C10—C9	119.6 (3)	C26—C27—H27B	108.9
C11—C10—H10	120.2	C26—C27—C28	113.23 (14)
C10—C11—H11	119.6	H27A—C27—H27B	107.7
C12—C11—C10	120.7 (3)	C28—C27—H27A	108.9
C12—C11—H11	119.6	C28—C27—H27B	108.9
C11—C12—H12	120.1	N2—C28—C27	112.40 (15)
C13—C12—C11	119.8 (2)	N2—C28—H28	106.5
C13—C12—H12	120.1	N2—C28—C29	111.11 (13)
C12—C13—H13	119.8	C27—C28—H28	106.5
C12—C13—C14	120.4 (3)	C29—C28—C27	113.22 (15)
C14—C13—H13	119.8	C29—C28—H28	106.5
C9—C14—C13	120.1 (3)	O1—C29—C28	109.65 (16)
C9—C14—H14	119.9	O1—C29—H29A	113.2 (12)
C13—C14—H14	119.9	O1—C29—H29B	108.3 (13)
N1—C15—N2	111.42 (15)	C28—C29—H29A	111.7 (12)
N1—C15—C16	121.31 (15)	C28—C29—H29B	107.3 (12)
N2—C15—C16	127.12 (15)	H29A—C29—H29B	106.5 (16)
			
N1—C7—C8—N2	−0.3 (2)	C10—C9—C14—C13	0.8 (3)
N1—C7—C8—C9	170.93 (19)	C10—C11—C12—C13	−0.5 (4)
N1—C15—C16—N3	−116.1 (2)	C11—C12—C13—C14	0.3 (4)
N1—C15—C16—C17	60.7 (3)	C12—C13—C14—C9	−0.5 (4)
N2—C8—C9—C10	77.2 (2)	C14—C9—C10—C11	−1.0 (3)
N2—C8—C9—C14	−102.9 (2)	C15—N1—C7—C6	178.44 (19)
N2—C15—C16—N3	68.7 (3)	C15—N1—C7—C8	0.6 (2)
N2—C15—C16—C17	−114.5 (2)	C15—N2—C8—C7	0.0 (2)
N2—C28—C29—O1	64.47 (19)	C15—N2—C8—C9	−172.28 (17)
N3—C16—C17—C18	−1.9 (3)	C15—N2—C28—C27	−72.9 (2)
C1—C2—C3—C4	1.1 (4)	C15—N2—C28—C29	55.2 (2)
C1—C6—C7—N1	147.0 (2)	C15—C16—C17—C18	−178.6 (2)
C1—C6—C7—C8	−35.7 (3)	C16—N3—C20—C19	−0.2 (4)
C2—C1—C6—C5	0.9 (3)	C16—C17—C18—C19	0.6 (4)
C2—C1—C6—C7	−177.3 (2)	C17—C18—C19—C20	0.8 (4)
C2—C3—C4—C5	−0.4 (5)	C18—C19—C20—N3	−1.0 (4)
C3—C4—C5—C6	−0.1 (5)	C20—N3—C16—C15	178.34 (19)
C4—C5—C6—C1	−0.2 (4)	C20—N3—C16—C17	1.7 (3)
C4—C5—C6—C7	178.2 (2)	C21—C22—C23—C24	−0.9 (4)
C5—C6—C7—N1	−31.3 (3)	C21—C26—C27—C28	108.7 (2)
C5—C6—C7—C8	146.0 (2)	C22—C21—C26—C25	0.0 (3)
C6—C1—C2—C3	−1.4 (4)	C22—C21—C26—C27	179.8 (2)
C6—C7—C8—N2	−177.9 (2)	C22—C23—C24—C25	0.6 (4)
C6—C7—C8—C9	−6.6 (4)	C23—C24—C25—C26	0.0 (4)
C7—N1—C15—N2	−0.6 (2)	C24—C25—C26—C21	−0.3 (3)
C7—N1—C15—C16	−176.46 (17)	C24—C25—C26—C27	179.95 (18)
C7—C8—C9—C10	−92.9 (3)	C25—C26—C27—C28	−71.5 (2)
C7—C8—C9—C14	87.0 (3)	C26—C21—C22—C23	0.6 (4)
C8—N2—C15—N1	0.4 (2)	C26—C27—C28—N2	−64.04 (19)
C8—N2—C15—C16	175.96 (18)	C26—C27—C28—C29	169.04 (15)
C8—N2—C28—C27	112.38 (18)	C27—C28—C29—O1	−167.93 (15)
C8—N2—C28—C29	−119.57 (18)	C28—N2—C8—C7	175.71 (16)
C8—C9—C10—C11	178.9 (2)	C28—N2—C8—C9	3.4 (3)
C8—C9—C14—C13	−179.1 (2)	C28—N2—C15—N1	−175.13 (17)
C9—C10—C11—C12	0.9 (4)	C28—N2—C15—C16	0.4 (3)

### Hydrogen-bond geometry (Å, º)

**Table:**

*D*—H···*A*	*D*—H	H···*A*	*D*···*A*	*D*—H···*A*
C27—H27*A*···N3	0.97	2.42	3.277 (2)	146
C22—H22···O1^i^	0.93	2.54	3.350 (3)	146
O1—H1···N1^ii^	0.85 (3)	1.94 (3)	2.789 (2)	174 (3)

Symmetry codes: (i) *x*−1, *y*, *z*; (ii) *x*+1/2, −*y*+3/2, −*z*+1.
